# Genomics of founders for conservation breeding: the Jasper caribou case

**DOI:** 10.1007/s10592-023-01540-3

**Published:** 2023-07-03

**Authors:** Maria Cavedon, Lalenia Neufeld, Laura Finnegan, Dave Hervieux, Anita Michalak, Agnes Pelletier, Jean Polfus, Helen Schwantje, Geoff Skinner, Robin Steenweg, Caeley Thacker, Jocelyn Poissant, Marco Musiani

**Affiliations:** 1 Deparment of Biological Sciences, University of Calgary, Calgary, AB T2N 1N4 Canada; 2 Jasper National Park of Canada, Parks Canada, Jasper, Canada; 3 fRI Research, 1176 Switzer Drive, Hinton, AB T7V 1V3 Canada; 4 Fish and Wildlife Stewardship Branch, Alberta Environment and Protected Areas, Grande Prairie, AB T8V 6J4 Canada; 5 Faculty of Veterinary Medicine, University of Calgary, Calgary, AB T2N 1N4 Canada; 6 Ministry of Land, Water and Resource Stewardship Northeast Region, 400-10003-110Th Avenue, Fort St. John, BC V1J 6M7 Canada; 7 Canadian Wildlife Service – Pacific Region, Environment and Climate Change Canada, 1238 Discovery Ave, Kelowna, BC V1V 1V9 Canada; 8 Wildlife and Habitat Branch, Ministry of Forests, Lands, Natural Resource Operations and Rural Development, Government of British Columbia, 2080 Labieux Road, Nanaimo, BC V9T 6J 9 Canada; 9 Dipartimento Scienze Biologiche Geologiche Ambientali, Università Di Bologna, Via Zamboni, 33 - 40126 Bologna, Italia

**Keywords:** Caribou, Genomics, Conservation breeding, Founders, National Parks, Recovery actions

## Abstract

Conservation breeding programs are increasingly used as recovery actions for wild animals; bringing founders into captivity to rear captive populations for future reintroduction into the wild. The International Union for the Conservation of Nature recommends that founders should come from genetically close populations and should have sufficient genetic diversity to avoid mating among relatives. Genomic data are highly informative for evaluating founders due to their high resolution and ability to capture adaptive divergence, yet, their application in that context remains limited. Woodland caribou are federally listed as a Species at Risk in Canada, with several populations facing extirpation, such as those in the Rocky Mountains of Alberta and British Columbia (BC). To prevent local extirpation, Jasper National Park (JNP) is proposing a conservation breeding program. We examined single nucleotide polymorphisms for 144 caribou from 11 populations encompassing a 200,000^2^ km area surrounding JNP to provide information useful for identifying appropriate founders for this program. We found that this area likely hosts a caribou metapopulation historically characterized by high levels of gene flow, which indicates that multiple sources of founders would be appropriate for initiating a breeding program. However, population structure and adaptive divergence analyses indicate that JNP caribou are closest to populations in the BC Columbia range, which also have suitable genetic diversity for conservation breeding. We suggest that collaboration among jurisdictions would be beneficial to implement the program to promote recovery of JNP caribou and possibly other caribou populations in the surrounding area, which is strategically at the periphery of the distribution of this endangered species.

**Supplementary Information:**

The online version contains supplementary material available at 10.1007/s10592-023-01540-3.

## Introduction

As the rate of biodiversity loss caused by anthropogenic activities dramatically increases, numerous species and populations are now extirpated or face the threat of extirpation (Pimm et al. 2014). Consequently, recovery actions like conservation breeding programs are sometimes used to reintroduce species to areas where they used to occur, or to augment populations (Seddon et al. 2007; Brichieri-Colombi and Moehrenschlager 2016; Bubac et al. 2019). Conservation breeding programs encompass the act of bringing rare or endangered animals into captivity with the aim of rearing captive populations for eventual reintroduction of their progeny into the wild (Seddon et al. 2014). Examples of such programs for ungulates include those conducted for the European bison (*Bison bonasus*) in Poland (Tokarska et al. 2009), the Przewalski’s horse in Mongolia and China (Der Sarkissian et al. 2015), the American bison (*Bison bison*) in Oklahoma (Kleiman 1989), USA, the Arabian oryx (*Oryx leucoryx*) in Arabia (Arif et al. 2010), and the Pére David's deer (*Elaphurus davidianu*) in China (Dayuan et al. 2022).

Despite multiple examples of successful conservation breeding programs, their execution is challenging (Fischer and Lindenmayer 2000; Armstrong and Seddon 2008). To improve their success, guidelines and best practices are continuously improved (Frankham 2010). For example, the International Union for the Conservation of Nature (IUCN) guidelines indicate that reintroductions and other conservation translocations should be based on plans that evaluate economic, social, and ecological aspects, such as a species’ current and historical distribution, and the removal or substantial reduction, of extinction threat(s) (IUCN/SSC 2013). The IUCN guidelines emphasize that selection of appropriate founders is a critical factor affecting reintroduction success (IUCN/SSC 2013; Forsman 2014). Ideally, founders should have appropriate demographic characteristics (e.g., number, sex, and age), come from geographically close populations, and have characteristics (i.e., ecology, behaviour, etc.) similar to the populations being restored (Robert 2009; Soorae 2018).

Considering the genetics of founders is crucial for the success of conservation breeding programs (Araki et al. 2007; Witzenberger and Hochkirch 2011). From a genetic perspective, the best source populations are those that are genetically similar to the populations that had been extirpated and have sufficient genetic diversity to reduce mating among relatives of captive individuals (Frankham 2010; Pelletier et al. 2009; Miller et al. 2010). However, recent work has also indicated that genetic diversity is important and matching genetic groups may be overstated if there is not a biological difference between the groups, i.e. (Frankham et al. 2017; Ralls et al. 2020). Accounting for proper genetic characteristics of founders can increase the survival probability of released individuals and improve the chance of having healthy and self-sustaining reintroduced populations in the long term (Robert 2009; Williams and Hoffman 2009).

Until recently, genetic studies aimed at identifying founders for conservation breeding programs have been based on small sets of putatively neutral molecular markers (i.e., regions of the genome that are non-coding) such as microsatellites and/or mitochondrial DNA (mtDNA) (Russello and Jensen 2018). However, these markers have known limitations, including limited resolution and ability to capture adaptive differentiation. Single nucleotide polymorphisms (SNPs), have the potential to overcome such limitations and be more informative for the selection of founders (Allendorf et al. 2010; He et al. 2016). The higher resolution of SNPs can provide more precise estimates of genetic parameters (e.g., genetic diversity and inbreeding) of candidate founders when compared to microsatellites and mtDNA (Luikart et al. 2003; Ivy et al. 2009; Galla et al. 2020). In addition, as SNPs may be located in genes, they can be used to identify specific loci of interest in founders that might be desirable to maintain (e.g., loci under selection) in the captive and reintroduced populations (Laikre 1999; Luikart et al. 2003; Weeks et al. 2011; Flanagan et al. 2018; Wright et al. 2021).

**Fig. 1:**
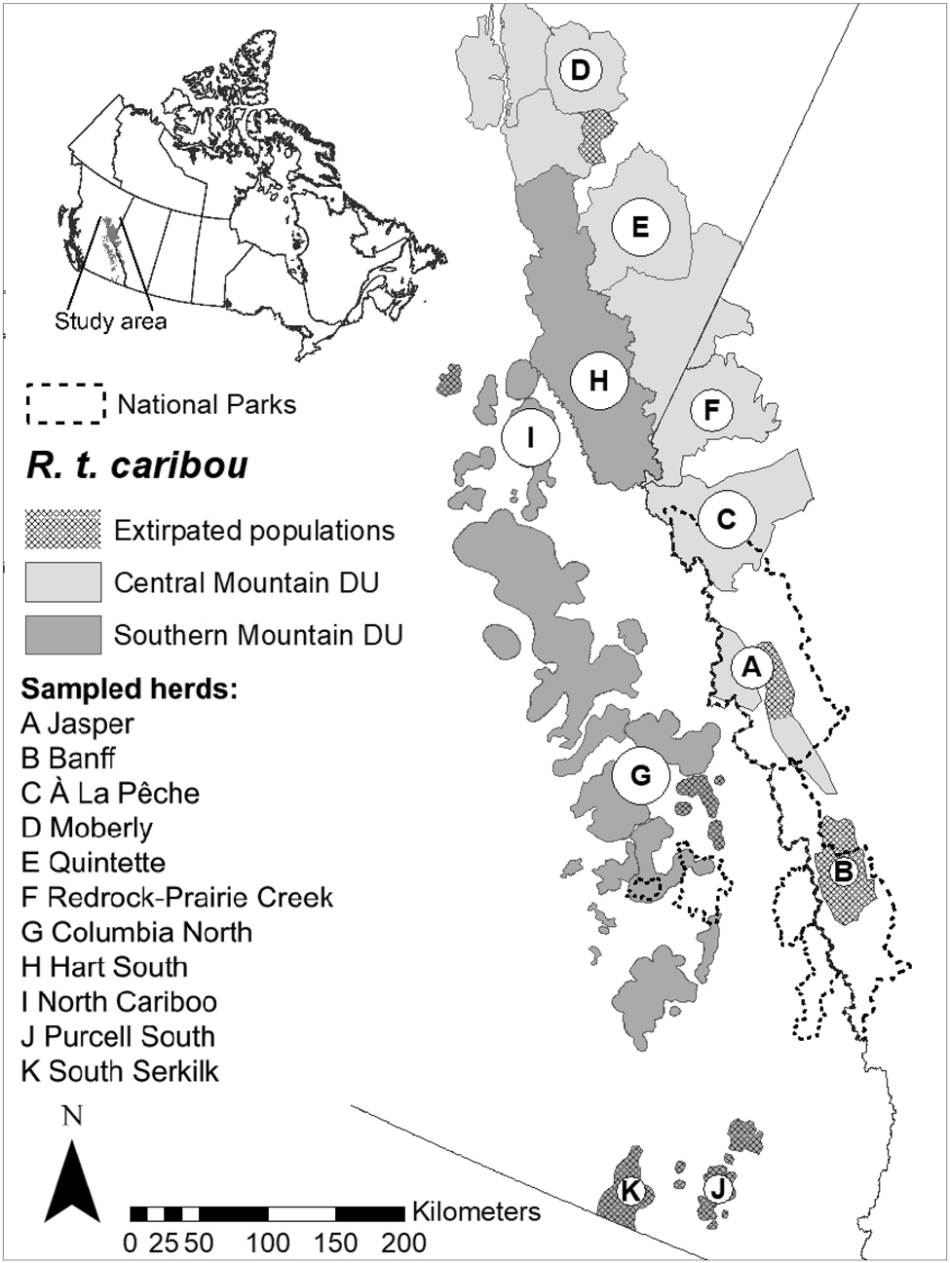
Caribou sampled in the southern Rocky Mountains of Canada for genomic analyses (*n* = 137; sampling locations in Fig. 3). Black lettered circles indicate sampled herds (i.e., a term used to indicate subpopulations, although herds of Woodland caribou are not social groups) with circle size proportional to sample size (mean = 12.45, SD = 7.03, range 2–20). Grey-scale polygons show the distribution of Designatable Units (DUs) encompassing multiple herds

Caribou (*Rangifer tarandus*), also known as reindeer, are a Holarctic species that is globally declining, and listed as a Species at Risk in Canada (Environment Canada 2014). Woodland caribou populations located at the southern edge of the species’ distribution, such as those in the study area in the Rocky Mountains of Alberta (AB) and British Columbia (BC) (Fig. 1), are particularly at risk of disappearing, also due to climate change (Vors and Boyce 2009). These caribou populations belong to one subspecies (Woodland Caribou) and two Designatable Units (DU; Central and Southern Mountain), which largely overlap in meaning with Evolutionary Significant Units (ESUs; Crandall et al. 2000; Green 2005; COSEWIC 2011). DUs are further divided into subpopulations, also known as “herds” (although herds of Woodland Caribou are not social groups; Bergerud 1988), delineated for management and conservation purposes (Environment Canada 2014).

In the southern Rocky Mountains of Canada, caribou herds occur on public lands available for multiple land uses including resource extraction, and in provincially and federally protected areas, however even in protected areas some have been extirpated and others are declining (Environment Canada 2014; Serrouya et al. 2019). In the mountain national parks, the last 9 caribou were extirpated from Banff in 2009 (Hebblewhite et al. 2010), and caribou now only occur in Revelstoke and Jasper (JNP) national parks. Caribou in JNP are either extirpated or facing near-extirpation, a status that calls for conservation actions (Parks Canada Agency 2022a, b). Caribou declines in JNP are due to multiple factors. Decades ago the JNP caribou lost migratory access to traditional forested foothills outside of the national park, due to high levels of habitat change and loss from industrial land uses. Loss of annual migration has been demonstrated to compromise the population viability of central mountain caribou (Williams et al. 2021). In addition, caribou remaining within JNP have been negatively affected by apparent competition with elk (Holt 1977) mediated by wolves (Bradley and Neufeld 2012). A rapid increase of elk density, which occurred after their reintroduction in 1960, resulted in higher wolf densities and an increase in predation rate on elk and caribou. Recently, lower wolf and elk densities have resulted in more favourable ecological conditions for caribou, and potential for recovery (Parks Canada Agency 2022a, b). Despite these current more favourable conditions, caribou within JNP continue to struggle with low survival rates, and the population is now too small to recover without population augmentation (Parks Canada Agency 2022a, b). Following IUCN guidelines, JNP is taking actions to aid recovery by exploring conservation breeding of JNP caribou. As all caribou herds within JNP are either extirpated, near extirpated or declining and as the habitat for caribou has been considered either favourable or improving, conservation breeding was considered a viable option by conservation planners and stakeholders (Foundations of Success and Parks Canada 2021; Salafsky et al. 2022). This exploration includes assessment of genetic information to identify options for selection of founder animals (Parks Canada Agency 2022a, b).

Caribou genetics studies conducted in the southern Rocky Mountains of AB and BC have primarily used neutral molecular markers (i.e., mitochondrial DNA haplotypes and autosomal microsatellites) (McDevitt et al. 2009; Serrouya et al. 2012; Weckworth et al. 2012 Yannic et al. 2014), although more recent work has used genomic data (Cavedon et al. 2019; Taylor et al. 2022). MtDNA indicated the presence of a hybrid zone where sympatric individuals have either Beringian/Northern or Southern mitochondrial lineage (McDevitt et al. 2009; Yannic et al. 2014). Studies based on microsatellites and SNPs also indicated the presence of shared genetic traits (McDevitt et al. 2009; Serrouya et al. 2012; Weckworth et al. 2012; Cavedon et al. 2019; Taylor et al. 2022). For example, population structure studies indicated that JNP caribou had genetic characteristics of both Central and Southern Mountain DU herds but that it was not possible to ascertain which characteristics predominated (McDevitt et al. 2009; Serrouya et al. 2012; Weckworth et al. 2012). However, past studies did not survey an adequate number of herds in the immediate JNP area to clarify local genetic structure and inform founder selection.

We conducted a genomic study to inform JNP’s planned conservation breeding program. We characterised genomic diversity in 144 individuals from 11 herds in a 200,000 km^2^ area using a newly developed caribou SNP array (Carrier et al. 2022), and sampling equally from the Central and Southern Mountain DUs. Our aims were to: (i) define levels of genetic differentiation, (ii) evaluate population structure, and (iii) detect signatures of adaptive divergence between population groups determined with population structure analyses. The goal of our study was to identify suitable founder populations for the establishment of a JNP conservation breeding program, and to interpret the findings in view of genetic and ecological traits of caribou in the southern Rocky Mountains of AB and BC.

## Materials and methods

### Sampling and genomic data

Blood and tissue samples were collected from both live and deceased animals as part of government agencies’ caribou monitoring activities between 2012 and 2020, corresponding to ≥ one generation for caribou (following McLoughlin et al. 2003).The collected samples spanned a 200,000 km^2^ study area located in the mountainous region along the southern AB and BC border in proximity to JNP, Canada (Fig. 1). Caribou in this area belong to either the Central Mountain DU or the Southern Mountain DU (COSEWIC 2011) and are from the montane cordillera ecozone, which includes habitats ranging from alpine tundra, to dense coniferous forests, to dry sagebrush and grasslands (Ecological Stratification Working Group 1995).

We extracted DNA from samples using the Qiagen kits following manufacturer protocols for both single spin columns (DNeasy Blood & Tissue Kit) and 96-well plates (QIAamp 96 DNA QIAcube HT kit). DNA samples were then quantified using either the BioTek Synergy LX Multimode Reader or the Thermo Fisher Qubit 3 Fluorometer following Thermo Fisher with Qubit and Quant-iT 1X dsDNA high sensitivity (HS) or broad range (BR) Kits. One hundred and forty-four samples that yielded ≥ 400 ng of DNA were selected for analyses. Samples were normalized to a quantity of 400 ng, dried on a Thermo Scientific Savant SpeedVac DNA 130 Integrated Vacuum Concentrator System, and shipped at room temperature to Genome Québec Ltd (Montréal, Québec) where genotyping using the Illumina Caribou 60 K SNP array (Carrier et al. 2022) was outsourced. The array accounts for SNPs evenly distributed across the entire genome (~ every 50 Kb) with known minor alleles across populations world-wide. In addition, a subset of SNPs was selected to represent rare and local alleles which could be used for ecotype and population assignments—information urgently needed for conservation planning.

We used PLINK v1.9 (Purcell et al. 2007) to perform data quality control, which included filtering out individuals and SNPs with call rates < 0.98 and SNPs with a minor allele frequency (MAF) < 0.01. After filtering there were 44,112 SNPs remaining and 137 individuals from both the Central Mountain DU (n_herds_ = 6, i_ndividuals_ = 77) and Southern Mountain DU (n_herds_ = 5, i_ndividuals_ = 60) (Fig. 1). For population structure analyses (below), we used PLINK to further exclude SNPs exhibiting strong linkage disequilibrium (“–indep-pairwise 50 5 0.5”) and those not in Hardy–Weinberg equilibrium (“–hwe 0.001”), leaving 36,053 SNPs for each individual (Purcell et al. 2007). In addition, for population structure analyses, we removed 42 animals which had a close relative in the dataset (28 and 14 belonging to the Central Mountain DU and Southern Mountain DUs, respectively) based on an identity by descent (IBD; –genome) degree of recent shared ancestry threshold of 0.25 (second-degree relatives) (see for example Kominakis et al. 2021).

### Assessing and comparing diversity of caribou 

We used the R package DARTR (Gruber et al. 2018) to estimate observed (Ho) and expected heterozygosity (He) and inbreeding coefficients (*F*_IS_, with 95% Confidence Intervals (CI) of bootstrap values) for each herd as well as pairwise fixation index values (*F*_ST_) between herds. We then used *F*_ST_ values to derive the number of migrants per generation (Nm; i.e., an estimate of gene flow; Wright 1931). The applied metrics of *F*_ST_ are sensitive to a species’ heterozygosity, and *F*_ST_ as well as the population STRUCTURE analyses (below) also to sample sizes. Due to low sample size, we therefore combined individuals from the Banff herd (*n* = 3) with those from JNP (*n* = 15). We also combined individuals from the Purcell South herd (*n* = 4) with those from South Selkirk herd (*n* = 3). The pooling of these samples was warranted due to known genetic similarities (McDevitt et al. 2009; Serrouya et al. 2012; Weckworth et al. 2012) and geographic proximity. We estimated a kinship matrix between caribou individuals using the R package POPKIN (Ochoa and Storey 2021), and from this, we also obtained the inbreeding coefficients for each caribou. To avoid a bias and potential underestimation of kinship and inbreeding, we used a SNP dataset including all caribou individuals (i.e., also including relatives), and where SNPs were filtered by LD and HWE as is standard practice.

### Population structure analyses

To assess isolation by distance (IBD), we performed a Mantel test to detect the potential correlation between the geographic and genetic distances calculated between individuals pairwise. We calculated the geographic distances with the function “distGeo” within the R package GEOSPHERE (Karney 2013), whereas we calculated the genetic distances with the function “gl.dist.ind” within the R package DARTR (Gruber et al. 2018). Lastly, we performed the mantel test with the function “gl.ibd” also within the dartR package.

To visualize patterns of population structure, we calculated pairwise Nei’s genetic distances (Nei 1972) between all individuals using the R package StAMPP v1.6.1 (Pembleton et al. 2013) and constructed a neighbour-joining tree based on the genetic distances using the R package APE 5.2 (Paradis et al. 2004). We also evaluated population structure using STRUCTURE v2.3.4*,* which uses a Bayesian iterative algorithm to place samples into clusters (K) whose members share similar patterns of genetic variation (Pritchard et al. 2000; Falush et al. 2003). We ran STRUCTURE with 20,000 burn-in iterations followed by 50,000 sampling iterations for K = 1 through 10 (Schweizer et al. 2016; Cavedon et al. 2022a, b, c). Each run was performed 10 times, and the ΔK statistic of Evanno et al. (2005) was calculated using STRUCTURE HARVESTER to help determine the most appropriate number of genetic clusters (Earl and von Holdt 2012). For comparison, we also assessed population structure with a maximum likelihood approach implemented in ADMIXTURE v1.3 and the most appropriate number of genetic clusters was detected by examining the cross-validation errors for K varying from 1 to 10 (Alexander et al. 2009). Lastly, as is common in genetic studies examining population structure (see for example Cavedon et al. 2022a, b, c), we performed a principal component analysis (PCA) to determine population groups using the program SMARTPCA within EIGENSTRAT v3.0 (Price et al. 2006).

### Detection of adaptive divergence between caribou population groups

We identified putatively adaptatively divergent SNPs between population groups determined with population structure analyses (see results) with BAYESCAN v.2.1 (Foll and Gaggiotti 2008). BAYESCAN tests whether population group-specific allele frequencies, measured by an *F*_*ST*_ coefficient, are significantly different from the allele frequency within the common gene pool. It also assigns a posterior probability (alpha) to a model in which selection explains the difference in allele frequencies better than a null model. A positive alpha indicates population group-specific directional selection, while a negative alpha suggests balancing or purifying selection. We ran analyses using a prior odd of 10, where SNPs were considered to be under selection when below the false discovery rate (FDR) threshold of 0.05.

## Results

### Genetic diversity and gene flow 

**Fig. 2:**
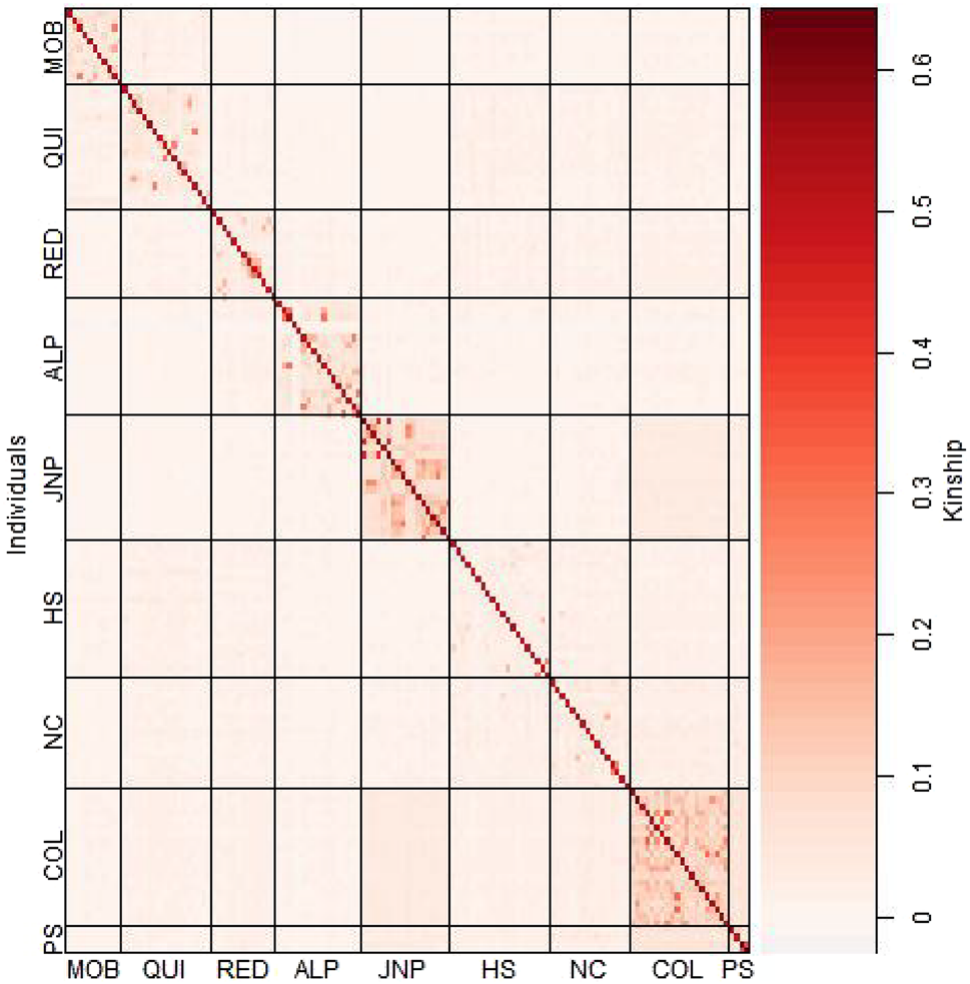
Kinship matrix of caribou individuals. Colors indicate the degree of relatedness between individuals (darker color indicate higher relatedness). Individuals were grouped by herd: MOB = Moberly; QUI = Quintette: RED = Redrock-Prairie Creek; ALP = À La Pêche: JNP = Jasper + Banff; HS = Hart South; NC = North Caribou; COL = Columbia North; PS = South Selkirk and Purcell South

Observed heterozygosity (Ho) of caribou herds in the study area ranged from 0.33 to 0.39 (mean 0.37 + SD 0.02; Table 1) and expected heterozygosity (He) ranged from 0.35 to 0.39 (mean 0.37 + SD 0.01). Inbreeding coefficients (*F*_IS_) ranged from 0.003 to 0.047 (mean 0.019 + SD 0.015). Pairwise fixation index (*F*_ST_) between herds ranged from 0.01 to 0.09 (mean 0.06 + SD 0.02). *F*_ST_ values between JNP and the other herds varied from 0.06 to 0.09 (mean 0.07 + SD 0.04; Table 2). The estimated number of migrants per generation (Nm values) between herds assessed pairwise were all > 1 (mean 5.38 + SD 4.10). The estimated Kinship matrix indicated moderate relatedness among caribou individuals within the same herd (Fig. 2), with the exception of caribou within the Hart South and North Caribou herds. The matrix indicated lower relatedness among caribou individuals belonging to different herds. The inbreeding coefficients for caribou individuals calculated from the kinship matrix followed a gaussian distribution (Fig. S1).

**Table 1:** Values of observed and expected heterozygosity (Ho and He respectively), and inbreeding coefficient (*F*_IS_, with Confidence Intervals in parenthesis) calculated using SNPs for caribou herds belonging to the Central Mountain or Southern Mountain DU, sampled in western Canada between 2012 and 2020

	Central Mountain DU					Southern Mountain DU			
	Jasper^a^	À La Pêche	Moberly	Quintette	Redrock	Columbia North	Hart South	North Cariboo	Purcell South^b^
Ho	0.35	0.38	0.39	0.38	0.39	0.36	0.39	0.39	0.33
He	0.36	0.38	0.38	0.39	0.38	0.35	0.39	0.39	0.36
*F*_IS_	0.028 (0.024, 0.032)	0.017 (0.014, 0.020)	0.029 (0.026, 0.032)	0.004 (0.002, 0.006)	0.021 (0.018, 0.024)	0.014 (0.012, 0.016)	0.004 (0.002, 0.006)	0.003 (0.001, 0.005)	0.047 (0.022, 0.072)

^a^Individuals from Banff and Jasper were combined

^b^Individuals from South Selkirk and Purcell South were combined (see “Methods” section)

**Table 2:** Values of pairwise fixation index (*F*_ST_) (below diagonal) and number of migrants per generation (Nm) (above diagonal) calculated using SNPs between caribou herds belonging to Central Mountain or Southern Mountain DU, sampled in western Canada between 2012 and 2020

Pairwise *F*_ST_ (Nm)	Jasper	À La Pêche	Moberly	Quintette	Redrock	Columbia North	Hart South	North Cariboo	Purcell South
Jasper^a^	–	2.88	2.53	3.32	3.32	2.88	3.92	3.92	2.53
À La Pêche	0.08	–	3.92	4.75	6	2.88	6	6	2.88
Moberly	0.09	0.06	–	0.04	0.05	0.09	0.04	6	2.88
Quintette	0.07	0.05	6	–	0.03	0.07	12.25	8.08	3.32
Redrock	0.07	0.04	4.75	8.08	–	3.32	12.25	8.08	3.32
Columbia North	0.08	0.08	2.53	3.32	0.07	–	3.92	3.92	3.92
Hart South	0.06	0.04	6	0.02	0.02	0.06	–	24.75	4.75
North Cariboo	0.06	0.04	0.04	0.03	0.03	0.06	0.01	–	4.75
Purcell South^b^	0.09	0.08	0.08	0.07	0.07	0.06	0.05	0.05	–

^a^Three individuals from Banff were added to the Jasper pool

^b^Two individuals from South Selkirk were added to the Purcell South pool (see “Methods” section)

### Population structure of caribou individuals

**Fig. 3:**
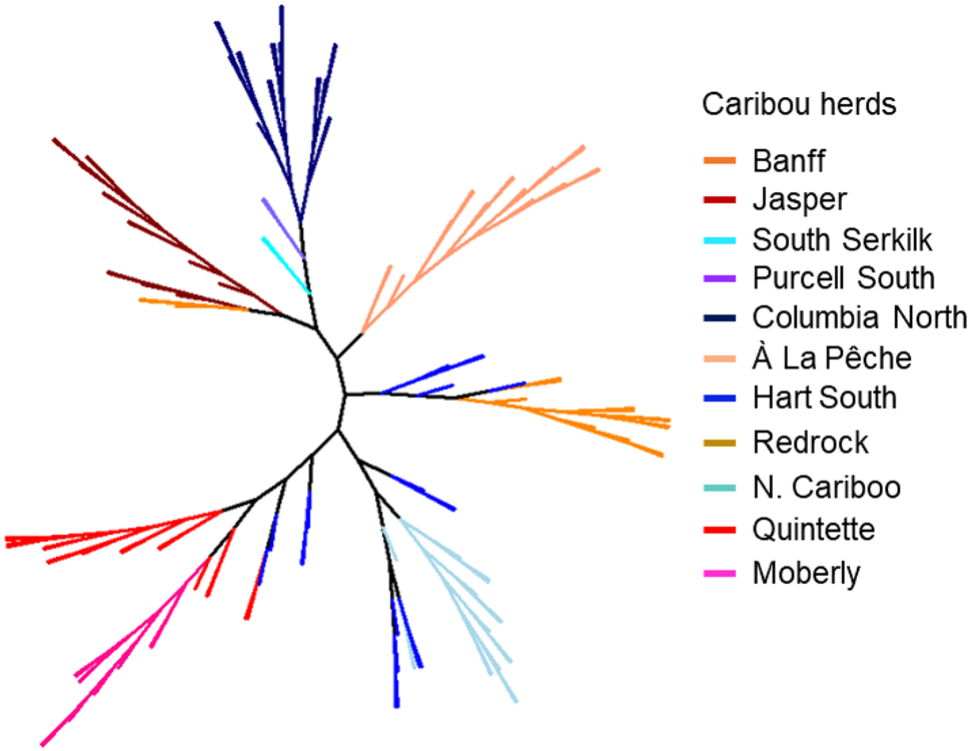
Neighbor-joining tree of caribou individuals sampled in the southern Rocky Mountains of Canada. The tree was based on Nei’s genetic distance between individuals calculated using genome-wide SNPs data. Branches represent caribou individuals and colors represent herds

We found a significant correlation between geographic distance and genetic distance, across the 137-individual SNP data set (*r* = 0.499; Mantel test *P* = 0.001; Fig. S2). JNP and Banff individuals grouped together in the neighbor-joining tree and were most similar to caribou from Columbia North, Purcell South, and South Selkirk. JNP and Banff individuals then grouped together with caribou from À La Pêche, and then caribou from Redrock-Prairie Creek (a group that was admixed with some Hart South individuals) (Fig. 3). The remaining groups (Hart South, North Cariboo, Quintette, and Moberly) were admixed and branched together separately from JNP and Banff (Fig. 3).

**Fig. 4:**
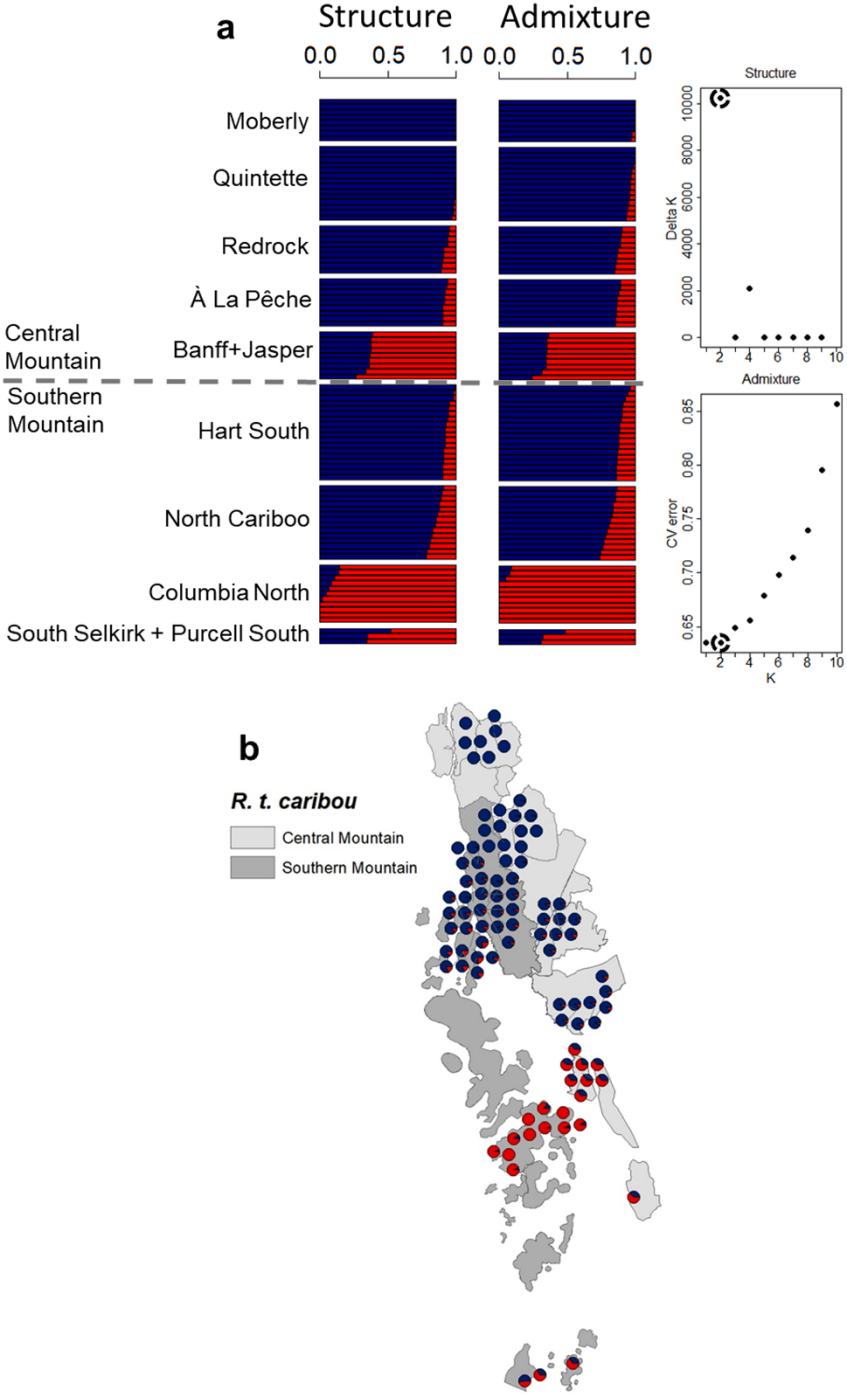
Population structure of caribou sampled in the southern Rocky Mountains of Canada. **a** Bar plots from STRUCTURE and ADMIXTURE analyses indicate individual proportions of assignment into two main genetic clusters (red or blue color). The most likely number of clusters (K) obtained with STRUCTURE (higher values best) and ADMIXTURE (lower values best) is indicated with circles on the respective scatter plots. **b** Map showing distribution of sampled caribou (capture coordinates) with proportion of belonging to either cluster for each individual obtained with STRUCTURE (pie chart)

STRUCTURE and ADMIXTURE analyses both supported the presence of two genetic clusters (*K* = 2, Fig. 4). Results also indicated that JNP and Banff individuals had similar assignment to individuals belonging to the Columbia North, Purcell South, and South Selkirk populations, but were different from individuals from À La Pêche, Redrock-Prairie Creek, Quintette, Moberly, Hart South, and North Cariboo.

**Fig. 5:**
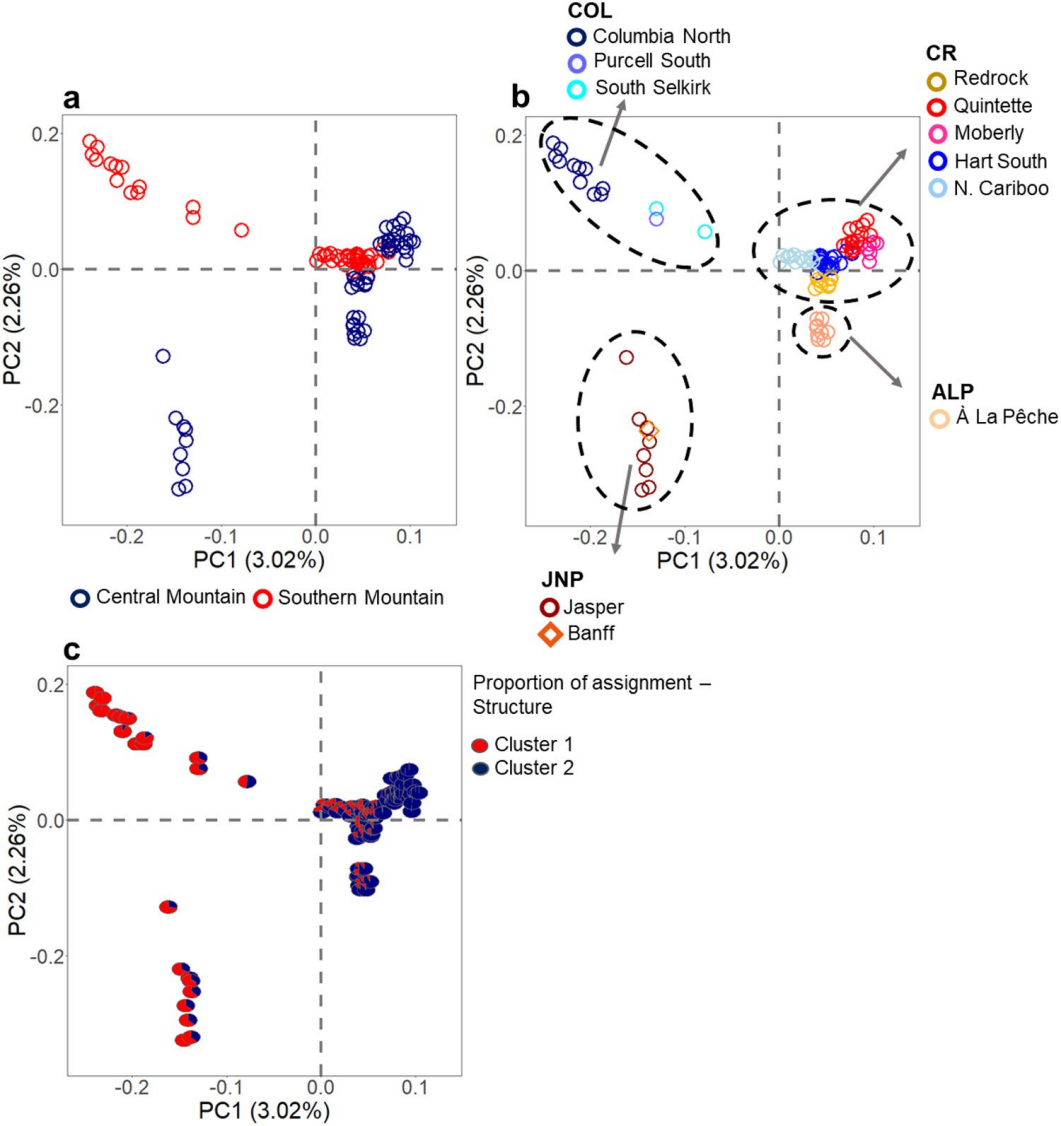
Principal Component Analysis (PCA) of caribou sampled in the southern Rocky Mountains of Canada. Circles represent caribou individuals, and colors represent DUs (in Panel **a**), herds (in Panel **b**) or proportion of assignment to the two major genetic clusters detected with STRUCTURE (in Panel **c**). Dashed circles in panel B indicate the caribou population groups we detected, which were then used in analyses of adaptive divergence: ALP = À La Pêche; COL = Columbia North, Purcell South, and South Selkirk; JNP = Jasper and Banff; CR = Moberly, Quintette, Redrock-Prairie Creek, Hart South, and North Cariboo

A principal component analysis indicated that individuals belonging to Central Mountain or Southern Mountain DUs could not be obviously separated along the two principal axes (Fig. 5a). It also indicated that some Central and Southern Mountain DU caribou were genetically close, including an assemblage formed by À La Pêche, Redrock-Prairie Creek, Quintette, Moberly, Hart South, and North Cariboo individuals, and including another assemblage formed by Columbia North, South Selkirk, Purcell South, JNP, and Banff individuals (Fig. 5b). JNP and Banff caribou could not be distinguished from Columbia North, Purcell South, and South Selkirk along the PC1 axis, whereas they could be distinguished from other caribou along the PC2 axis. The first and second axis accounted for 3.37 and 2.79% of the observed genetic variation, respectively (the 3rd axis accounted for 1.88%). Our analyses therefore determined four distinguishable groups (Fig. 5b), including caribou individuals with varying proportions of assignment to the two major genetic clusters detected with STRUCTURE (Fig. 5c). A population group (called “JNP”) was formed by individuals from the JNP and Banff herds. One more population group was formed by individuals from À La Pêche (“ALP”), one additional by individuals from Columbia North, Purcell South, and South Selkirk (“COL”). The fourth and final population group (“CR”) included individuals from the remaining sampled herds, which were also close on the PCA plot (Hart South, North Cariboo, Redrock-Prairie Creek, Quintette, and Moberly).

### Divergence between caribou population groups

**Fig. 6:**
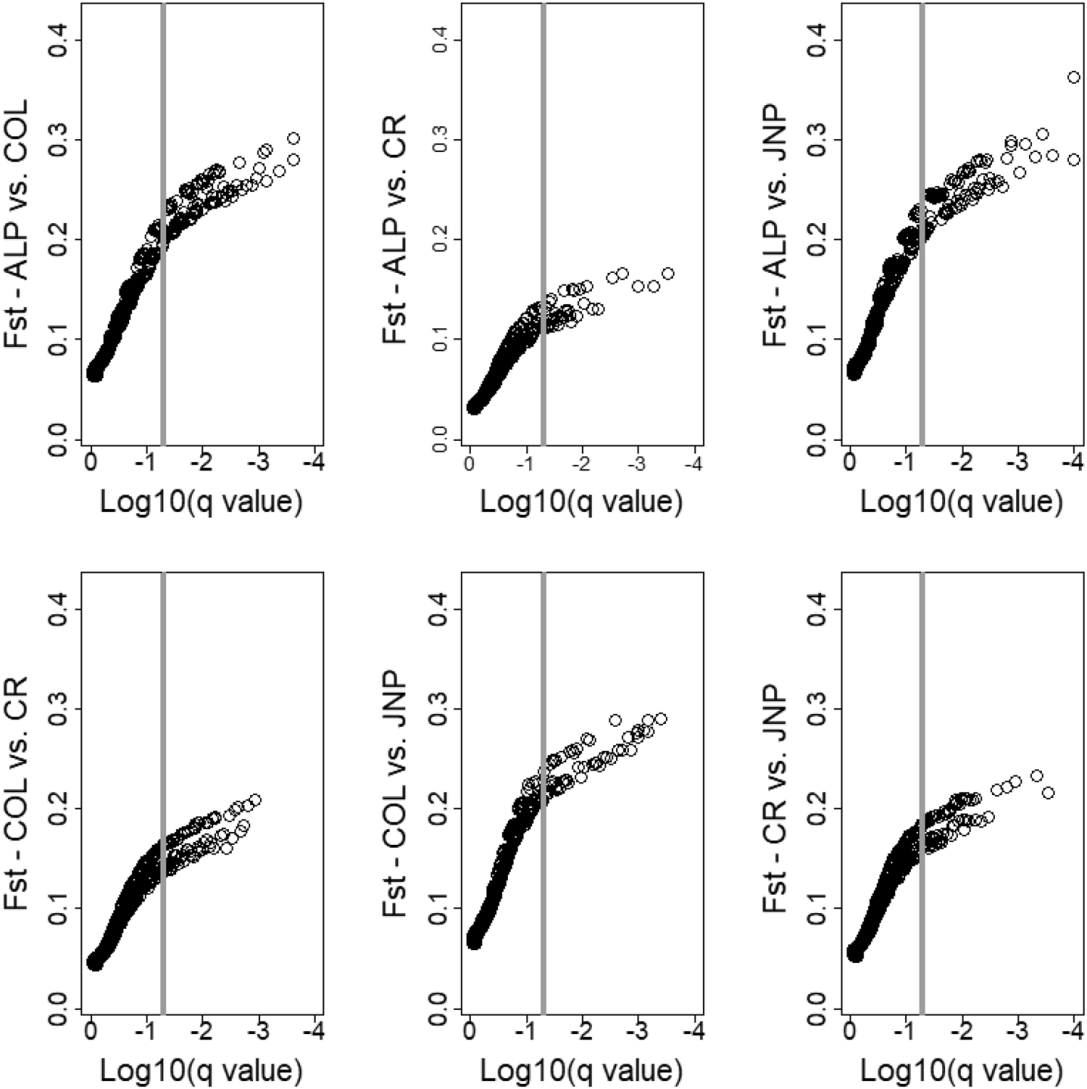
Signatures of adaptive divergence between caribou population groups determined by outlier analyses. The horizontal axis indicates the BAYESCAN*-*assessed log10 of the *q* value (the false discovery rate (FDR) analog to the *p*-value) and the vertical axis is the mean genetic differentiation (*F*_*ST*_). Each point represents a SNP and significant outliers are visible right of the grey vertical line. Identifiers represent population groups: ALP = À La Pêche; COL = Columbia North, Purcell South, and South Selkirk; JNP = Jasper and Banff; CR = Moberly, Quintette, Redrock-Prairie Creek, Hart South, and North Cariboo

To assess putatively adaptive divergence, we ran pairwise BAYESCAN analyses between the four caribou population groups identified with PCA analyses (see above). We identified 89 outlier SNPs divergent between JNP and ALP, with *F*_*ST*_ values ranging between 0.20 and 0.36 (average 0.25). We also detected 68 SNPs diverging between JNP and CR, with *F*_*ST*_ values ranging between 0.15 and 0.24 (average 0.19). Lastly, we found only 53 outlier SNPs between JNP and COL with *F*_*ST*_ values ranging between 0.21 and 0.29 (average 0.25; Fig. 6).

## Discussion

We conducted a genomic study to identify candidate founders for a conservation breeding program currently planned for caribou, an approach suggested as a best practice for endangered species in general, but requiring substantial preparation and research to be successful (Russello and Jensen 2018). Our work relied on a newly developed SNP-array specifically produced for caribou (Carrier et al. 2022), which in this study produced around 40,000 SNPs that were successfully genotyped in 95% of the sampled individuals. Across the 200,000 km^2^ study area we found that, despite wide-spread recent declines (Serrouya et al. 2019), caribou subpopulations (herds) have retained levels of genetic diversity and natural connectivity (e.g. more than one migrant per generation [Wright 1931; Slatkin 1987; Weeks et al 2011] genetically estimated by this study between any pair of herds) which in other studies have been considered adequate for conservation interventions including translocations of individuals. However, we found two major genetic clusters and additional population groups, which should inform conservation planning. If a conservation breeding program is established in JNP, founders could be selected from clusters or population groups most similar to JNP as a first priority, while also taking into consideration information on potentially-adaptive divergence (see discussion below on results of outlier analyses).

The levels of heterozygosity we identified (including He and Ho) were aligned with other reported values for caribou and approaching levels considered as “high” in wild mammals (see for example Cavedon et al. 2019). The levels of inbreeding coefficients we also determined (*F*_IS_), which provide information on relatedness among individuals (Crow and Kimura 1970; Caballero et al. 2021), were low to moderate (see Solmundson et al. 2020). Therefore, our findings indicate that the study area’s wild caribou have retained levels of genetic diversity, which if properly maintained in captive populations could perhaps circumvent risk of inbreeding depression. Also valuable to avoid inbreeding depression, in this study, we identified close relatives (those with IBD > 0.25 corresponding to second-degree relatives), which should be discarded when selecting source founders. Kinship is an important tool used to identify breeders for captive populations and can be used to effectively evaluate the potential for future inbreeding in wild populations (Ballou and Lacy 1995; Frankham et al. 2017; Flesch et al. 2022). Our kinship findings indicated moderate relatedness only among caribou individuals within the same herd and suggested that inbreeding could be minimized by relying on founders from different source herds, should a captive population be established. Our results also indicated that herds known to overlap (example, Hart South and North Caribou with other neighbouring herds; Environment Canada 2014) naturally had lower relatedness. Future studies could examine inbreeding and relatedness in more detail, such as by examining “runs of homozygosity” (see Broman and Weber 1999) across the genome, an approach that may show signatures of natural and/or human mediated selection (Kardos et al. 2015; Caballero et al. 2021; Solmundson et al. 2020).

We estimated that the number of migrants per generation (Nm) were all greater than one, indicating significant historical and/or recent gene flow among all herd pairs. One migrant per generation, as a minimum, is typically considered sufficient to offset genetic deterioration within subunits (Wright 1931; Slatkin 1987; Weeks et al 2011). It is therefore likely that caribou herds in the study area were connected until recently, with barriers to gene flow likely arising in the last decades. Consistent with this interpretation, barriers to dispersal have been identified in studies of radio-collared southern mountain caribou (Van Oort et al. 2011), which likely reflect contemporary, non-historical patterns (i.e., those observed during the 2–3 years lifespan of a GPS collar), and which were deployed just on females (i.e., the least vagile sex in caribou, as discussed in Cavedon et al. 2022a, b, c). By contrast, presence of barriers between population ranges was not as consequential: habitat suitability followed by predation risk was associated with overall gene flow in a caribou study conducted previously (Gubili et al. 2017). Importantly,, levels of Nm exceeding one have been historically used to manage wildlife populations as one unit (Mills and Allendorf 1996; Vucetich and Waite 2000; Wang 2004), and perhaps the same threshold could be applied to caribou (manageable in the future as a unit, similar to other wildlife populations). The levels of Nm we detected could be suitable for conservation translocations among these populations. This study’s populations are also within natural dispersal distances characteristic of the species, whereas dispersals themselves might be infrequent recently, likely due to habitat fragmentation (Van Oort et al. 2011). Nonetheless, further analyses on gene flow are recommended, as both *F*_ST_ and Nm are known to also depend on genetic diversity and on sample sizes (Holsinger and Weir 2009), which were limited in this study.

Population structure results indicate that caribou individuals could be grouped in two major genetic clusters, which is consistent with both past and current studies conducted in western Canada (see McDevitt et al. 2009; Cavedon et al. 2022a, b, c). In this study, we targeted common variants (SNPs with MAF > 0.01), which are suited to examine the deep evolutionary history of species (Gibson 2012). Our findings may therefore have captured similar diversification patterns as those detected with mtDNA analyses, which indicated the presence of either a Beringian/Northern or a Southern lineage in the study area (McDevitt et al. 2009; Serrouya et al. 2012; Weckworth et al. 2012; Yannic et al. 2014; Taylor et al. 2021).

The two genetic clusters we identified did not overlap fully with currently recognized DUs. We found that although caribou from some Central Mountain DU herds were genetically similar to one another, they were also similar to caribou from some Southern Mountain herds (note the mainly blue circles in Fig. 4b), and this information could also be used to plan future translocations. Our findings indicating the presence of two genetic clusters and potentially two DUs in the area are consistent with other recent studies also relying on genomic data for caribou (Cavedon et al. 2022a, b, c; Taylor et al. 2020, 2021). Our findings, however, partially contrast with past studies that rely on neutral markers (autosomal microsatellites and mtDNA), which have also indicated the presence of two main clusters and DUs, but with slightly different boundaries (McDevitt et al. 2009; Serrouya et al. 2012; Weckworth et al. 2012). This can be explained by the fact that these neutral genetic markers cannot detect local adaptation, unlike genomic SNPs (Luikart et al. 2003; Allendorf et al. 2010).

The principal component analysis detected four distinguishable groups, which we used for further analyses of potentially adaptive divergence. Overall, we found more outlier SNPs between JNP and ALP than between JNP and CR and especially between JNP and COL. Outlier SNPs may indicate divergence due to demographic events or selection processes related to local adaptation (Luikart et al. 2003; Allendorf et al. 2010). Previous genomic studies conducted for caribou on a larger scale also identified candidate loci under selection that were associated with ecological, behavioral, and climatic factors (Cavedon et al. 2019, 2022a, b, c). Together, the results of our current and previous studies (Cavedon et al. 2019, 2022a, b, c) indicate that caribou population groups in the study area may be differently adapted due to selective forces (e.g., environmental and climatic conditions), an important detail that should be accounted for in any conservation plan (Des Roches et al. 2021).

Our study of caribou genomics and distribution should inform selection of founders for the proposed JNP conservation breeding program. We found that the study area likely hosted a historic caribou metapopulation, characterized by high levels of gene flow which has led to high levels of genetic diversity. Our results showed that JNP could potentially acquire founders from any of the sampled herds, some of which, thanks to recovery actions, have recently started to increase and might be able to sustain the removal of some individuals (Government of Alberta 2017; Eacker et al. 2019; McNay et al. 2022). However, population structure analyses and adaptive divergence analyses indicate that JNP caribou are most genetically related to caribou in the Columbia range of BC. Selecting founders from the Columbia range would provide the best chance of maintaining genetic traits most similar to JNP caribou. Herds in the Columbia range are however numerically low compared to others (e.g., 184 individuals in Columbia North vs. 405 in Hart; see also Serrouya et al. 2021; McNay et al. 2022) and might not tolerate the removal of individuals. However, admixture of two caribou genetic clusters has also been consistently documented throughout the study area (see McDevitt et al. 2009, this study), therefore indicating that any conservation program should aim at maintaining such diversity (sensu Frankham et al. 2017, Ralls et al. 2020) too. It should also be taken into consideration that caribou genetic diversity in the study area is correlated with ecological and behavioural diversity also including seasonal migration (McDevitt et al. 2009; Cavedon et al. 2022a, b, c), and any conservation program should aim at maintaining all diversities in these partially migrating populations (Cavedon et al. 2019).

In addition to genetic analyses, it will be important to conduct population viability analyses, or an equivalent assessment approach, to understand the conservation implications of caribou removals for source populations of this species at risk (Hoban et al. 2012).

Similarly to caribou, other endangered species can benefit from the evaluation of genomic data applied to conservation breeding programs (Russello and Jensen 2018), as a key step to identify suitable units or groups for sourcing founder animals beyond arbitrary, human-created boundaries. Preserving and restoring such intraspecies diversity, though the path we indicated for caribou, is key to maintaining a species’ evolutionary potential, and in turn its critical ecological functions (Des Roches et al. 2021).

### Supplementary Information

Below is the link to the electronic supplementary material.

Supplementary file1 (DOCX 67 KB)

## Data Availability

Caribou sample locations and population and DU range maps are available from the Government of British Columbia –see https://catalogue.data.gov.bc.ca/dataset/caribou-herd-locations-for-bc. Restrictions apply to the availability of this Species At Risk’s raw SNP data, which were used under license for this study’s academic personnel.
