# Supplementary material for: Genomics of founders for conservation breeding: the Jasper caribou case
Source: Conserv Genet. 2023 Jul 3;24(6):855–67. doi: 10.1007/s10592-023-01540-3 (PMC10638200; doi:10.1007/s10592-023-01540-3)
Supplement: Supplementary file 1 — Supplementary file1 (DOCX 67 KB) [file 10592_2023_1540_MOESM1_ESM.docx]

# Supplementary Information

# Genomics of Founders for Conservation Breeding: The Jasper Caribou Case

Maria Cavedon${}^{1}$, Lalenia Neufeld${}^{2}$, Laura Finnegan${}^{3}$, Dave Hervieux${}^{4}$, Anita Michalak${}^{5}$, Agnes Pelletier${}^{6}$, Jean Polfus${}^{7}$, Helen Schwantje${}^{8}$, Geoff Skinner^2^, Robin Steenweg${}^{7}$, Caeley Thacker${}^{8}$, Jocelyn Poissant${}^{5}$, Marco Musiani${}^{9}$

^1^${}^{1}$ ${}^{1}$*Dept. of Biological Sciences, University of Calgary, Calgary, AB T2N 1N4, Canada.*

*^2^*${}^{1}$ *Jasper National Park of Canada, Parks Canada, Jasper, Canada.*

*^3^*${}^{1}$ ${}^{1}$*fRI Research, 1176 Switzer Drive, Hinton, Alberta, T7V 1V3*

^4^${}^{1}$ *Fish and Wildlife Stewardship Branch, Alberta Environment and Protected Areas, Grande Prairie, AB T8V 6J4, Canada.*

^5^ *Faculty of Veterinary Medicine, University of Calgary, Calgary, AB T2N 1N4, Canada.*

*^6^ Ministry of Land, Water and Resource Stewardship Northeast Region, 400-10003-110th Avenue, Fort St. John, BC, V1J 6M7*

${}^{1}$^7^${}^{1}$ *Canadian Wildlife Service – Pacific Region, Environment and Climate Change Canada,1238 Discovery Ave, Kelowna, BC V1V 1V9, Canada.*

^8^${}^{1}$ *Wildlife and Habitat Branch, Ministry of Forests, Lands, Natural Resource Operations and Rural Development, Government of British Columbia, 2080 Labieux Road, Nanaimo, BC, V9T 6J 9, Canada.*

^9^${}^{1}$ ${}^{10}$*Dipartimento Scienze Biologiche Geologiche Ambientali, Università di Bologna, Via Zamboni, 33 - 40126 Bologna, Italia.*

^*^Corresponding author: Marco Musiani; e-mail: [marco.musiani@unibo.it](mailto:mmusiani@ucalgary.ca)

# Supplementary Figures

## Fig. S1


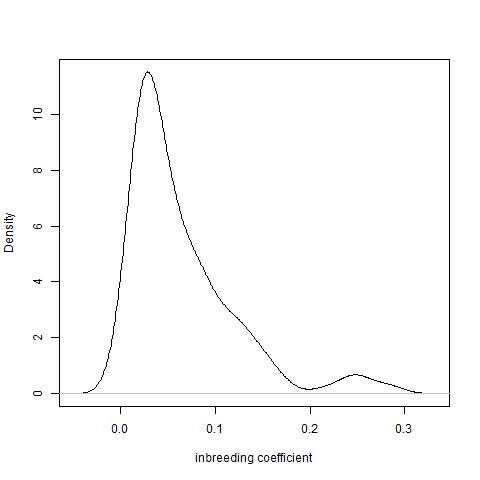


**Fig. S1** Density plot of inbreeding coefficients for 137 caribou sampled in the southern Rocky Mountains of Canada. Coefficients for each caribou individual were derived from kinship estimates.

## Fig. S2


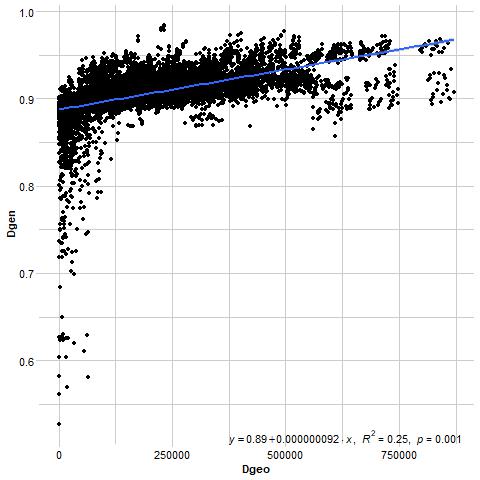


**Fig. S2** Isolation by distance for 137 caribou in the southern Rocky Mountains of Canada. Pairwise Genetic distances (Dgen) are plotted against the log10 of Euclidean geographic distance (Dgeo) among individuals sampled.
